# Kesterite-based optoelectronic synaptic memristors: a mini-review on material design and neuromorphic application

**DOI:** 10.1080/14686996.2026.2688751

**Published:** 2026-06-19

**Authors:** Fengxia Yang, Hao Sun, Xiaofei Dong, Xiang Zhang, Jiangtao Chen, Xuqiang Zhang, Yun Zhao, Yan Li

**Affiliations:** College of Physics and Electronic Engineering, Northwest Normal University, Lanzhou, China

**Keywords:** Kesterite, memristor, performance regulation, artificial synapse, neuromorphic application

## Abstract

With the rapid development of big data and artificial intelligence, the conventional Von Neumann architecture faces increasingly prominent issues of separation of storage and computing as well as high energy consumption, which severely hinder the progress of next-generation intelligent computing. Memristors, serving as fundamental components that enable in-memory computing and brain-inspired neuromorphic computing, naturally possess intrinsic benefits, namely high-density storage, smoothly adjustable conductance, and low power consumption, thus emerging as promising solutions to break through this bottleneck. Among various resistive switching materials, kesterite-based chalcogenides have become the promising choice for optoelectronic synaptic memristors due to their superior optoelectronic characteristics, rich elemental availability, green nature, and economic viability. This work concisely summarizes the research progress of kesterite-based optoelectronic synaptic memristors in terms of resistive switching performance optimization, synaptic behavior simulation, and neuromorphic applications, focusing on the effects of resistive switching layer thickness, elemental composition, heterojunction and composite structures, electrode engineering, as well as testing environment and operating modes. Furthermore, it discusses the current challenges and future development trends, providing an important guideline for designing, optimizing, and practically deploying high-performance neuromorphic devices based on kesterite.

## IMPACT STATEMENT

This review comprehensively summarizes various optimization strategies (thickness, composition, structure, electrode, environment, operation mode), synaptic behavior simulation, neuromorphic applications, and key challenges for emerging kesterite-based optoelectronic synaptic memristors.

## GRAPHICAL ABSTRACT

**Figure:**
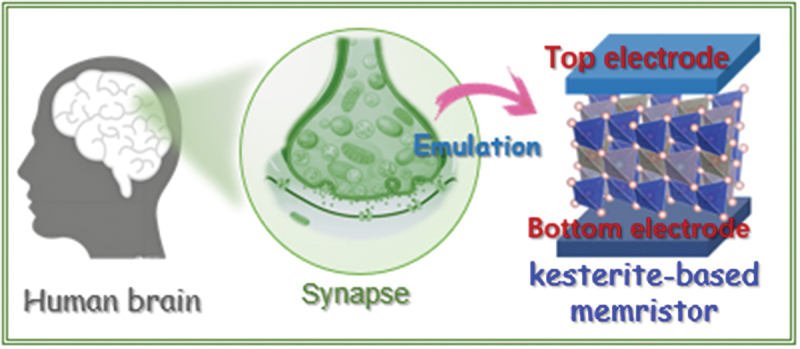

## 1. Introduction

With the advent of the artificial intelligence and big data era, traditional computing architectures face dual bottlenecks in energy efficiency and computational power due to the ‘memory wall’ problem [1–4]. The human brain, as a paradigm of natural information processing, employs a highly parallel, fault-tolerant, and low-power ‘in-memory computing’ architecture that efficiently handles complex and unstructured information such as image recognition and language comprehension, providing important insights for overcoming the limitations of the Von Neumann architecture [5–8]. In this architecture, synapses not only are connection nodes between neurons but also serve as memory units with learning capability: their connection strength can be dynamically modulated by neural activity, thereby enabling parallel information processing and adaptive learning [9–11]. Memristors, as two-terminal devices, whose resistance varies with the amount of charge that has flowed through them and retains its state nonvolatilely, possess continuously tunable conductance states under external stimuli, which can be used to precisely mimic the connection weights of biological synapses [12–14]. Owing to their highly structural and physical similarity to biological synaptic functions, memristors are widely regarded as key hardware building blocks for implementing artificial synapses and constructing large-scale neuromorphic computing systems [15–18].

The typical structure of a memristor is a sandwich‑type ‘metal‑insulating layer‑metal’ configuration, whose performance largely depends on the synergistic effect of the intermediate resistive switching layer and the two metal electrodes [19–21]. Among them, the intrinsic properties of the resistive switching layer (such as defect type, ion mobility, and band structure) together with the interfacial effects between the electrodes and the resistive switching layer directly determine key device performance parameters, including the On/Off ratio, endurance, and power consumption [22,23]. To achieve high‑performance optoelectronic synaptic memristors suitable for neuromorphic computing, researchers have systematically explored a variety of resistive switching material systems, including metal oxides (e.g. TiO_2_, WO_3_, Ga_2_O_3_) [24–26], nitrides (e.g. h‑BN, GaN) [27,28], two‑dimensional materials (e.g. MoS_2_, MoSe_2_, MXene) [14,29,30], organic polyelectrolytes (e.g. PTH-Fc) [31], perovskite materials (e.g. MAPbI_3_, Cs_3_Sb_2_I_9_) [10,32–34], and chalcogenides (e.g. PbS, Sb_2_Se_3_) [35–37]. Furthermore, a series of strategies such as constructing heterojunctions (e.g. CeO_2_/MoS_2_, V_2_C/V_2_O_5-x_, Bi_2_Te_3_/SnSe, MoS_2_/Te, Ta_2_O_5_/HfO_2_, ZnO/MoS_2_, IGZO/WO_3-x_) [38–45], adjusting the stoichiometric ratio of the resistive switching layer, and optimizing electrode material matching (e.g. Au, Ag, Mo, Pt, Al, AZO, FTO, ITO, TiN) can effectively modulate ion migration behavior and charge transport mechanisms within the resistive switching layer, thereby significantly improving the controllability and reliability of device performance [42,43,46–50].

Among various synaptic memristor devices, optoelectronic synaptic memristors with photoelectric co-modulation capability have attracted widespread attention due to their ability to mimic the integrated information processing mechanism of perception, storage, and computation in biological visual systems. Approximately 80% of the external perceptual information in humans originates from the visual system; therefore, developing neuromorphic hardware with photoresponsive characteristics is of great significance for constructing efficient bioinspired vision systems [38,51,52]. Such devices typically rely on functional materials that combine broadband spectral response and dynamic optoelectronic coupling properties. Multinary chalcogenides (e.g. CuInS_2_, AgInSbTe, CuInP_2_S_6_) have demonstrated good potential as resistive switching materials owing to their tunable bandgap and excellent light absorption capability [53–55]. Among them, kesterite-structured materials such as Cu_2_ZnSnSe_4_ (CZTSe), Cu_2_ZnSnS_4_ (CZTS), and Cu_2_ZnSn(S,Se)_4_ (CZTSSe) possess a unique combination of attributes that underscore their core value for optoelectronic synaptic memristors: they are composed entirely of earth‑abundant and non‑toxic elements, offering a sustainable and low‑cost solution; their direct bandgap can be finely tuned from 1.0 to 1.5 eV by adjusting the S/Se ratio, enabling optimal matching with the visible to near‑infrared spectrum; and they exhibit a high optical absorption coefficient (>10^4^ cm^−1^) [56,57]. In recent years, researchers have systematically investigated the performance regulation of kesterite-based memristors and have achieved a series of important advances in optically and electrically induced synaptic plasticity simulation as well as advanced neuromorphic functions [58–70].

**Figure 1.**
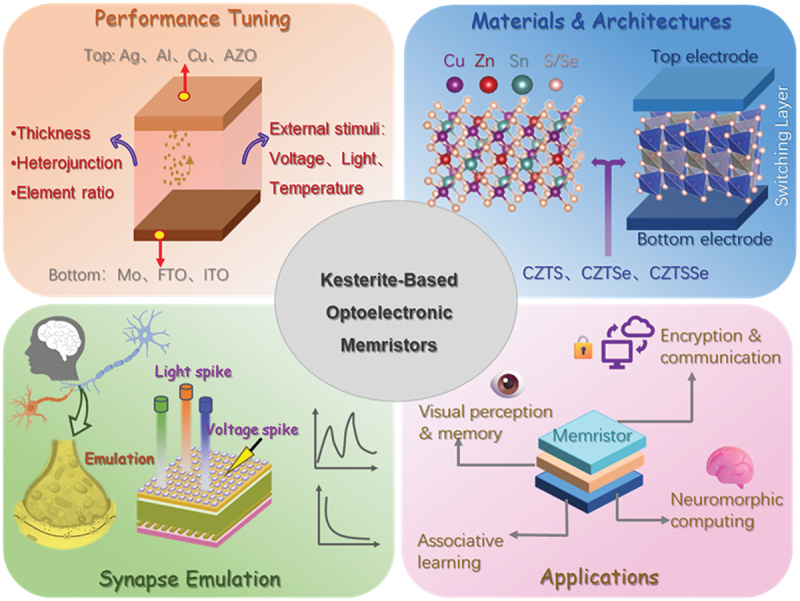
Schematic summary of kesterite‑based memristors.

This article briefly reviews the regulatory effects of key parameters such as resistive switching layer thickness, elemental ratios, heterojunctions and composite structures, electrode engineering, testing environment, and operating mode on the performance of kesterite‑based memristors. It also summarizes the research progress in optoelectronic synaptic plasticity and the applications of such devices in advanced neuromorphic functions, including information encryption and secure communication, neuromorphic computing and pattern recognition, as well as visual perception and associative learning. Finally, it provides an outlook on the challenges and future development directions of kesterite‑based memristors. Figure 1 summarizes the above content, and Table 1 lists the key device parameters.

**Table 1.** Key characteristics and applications of typical kesterite‑based memristors.

Device structure	On/Off ratio	Retention/Endurance	Synaptic	Applications	Ref
AZO/CZTSe/FTO	6.855	–		-	[58]
Al/CZTS-PMMA/FTO	32	200/10^4^	EPSC, PPF, LTP/LTD, STDP, STM/LTM	✓	[60]
Ag/CZTSSe/Mo	1027	120/10^4^	LTP/LTD, STM/LTM, classical conditioning	✓	[61]
Cu,Ag,Al/CZTS/ITO	32	100/10^3^	–	–	[67]
Al/CZTSSe/Mo	27.5	200/-	–	–	[62]
Ag/CZTS/ZnO/ITO	–	–	EPSC, PPF, classical conditioning	✓	[64]
Ag/BFO/CZTSe/FTO	22.4	500/10^4^	–	–	[65]
Al/CZTS:PMMA/ITO	10^9^	10^5^/-	–	–	[66]
Ag/CZTSe/Mo	215	200/10^4^	–	–	[68]
Cu/CZTS/ITO	3	1200/5000	PPF, STDP	–	[70]
Ag/CZTSSe/Mo	>10^3^	160/10^4^	EPSC, PPF, STP/STD, STDP	✓	[69]

## 2. Device parameter optimization

### 2.1. Thickness regulation of resistive switching layer

In the process of performance optimization and structural design of memristors, the thickness of the resistive switching layer, as an indispensable core structural parameter, plays a critically regulatory role in all aspects of device operation [11]. It directly governs the dynamic behaviors of conductive filament formation, growth, and rupture, modulates the transport pathways and migration efficiency of charge carriers, and simultaneously influences the width and height of the interfacial barrier between the electrode and the resistive switching layer [59]. Ultimately, it determines key performance metrics such as the On/Off ratio, operating voltage, and cycling stability, serving as one of the crucial regulatory means to achieve high performance and practical applicability of the device.

#### 2.1.1. CZTSe switching films

**Figure 2.**
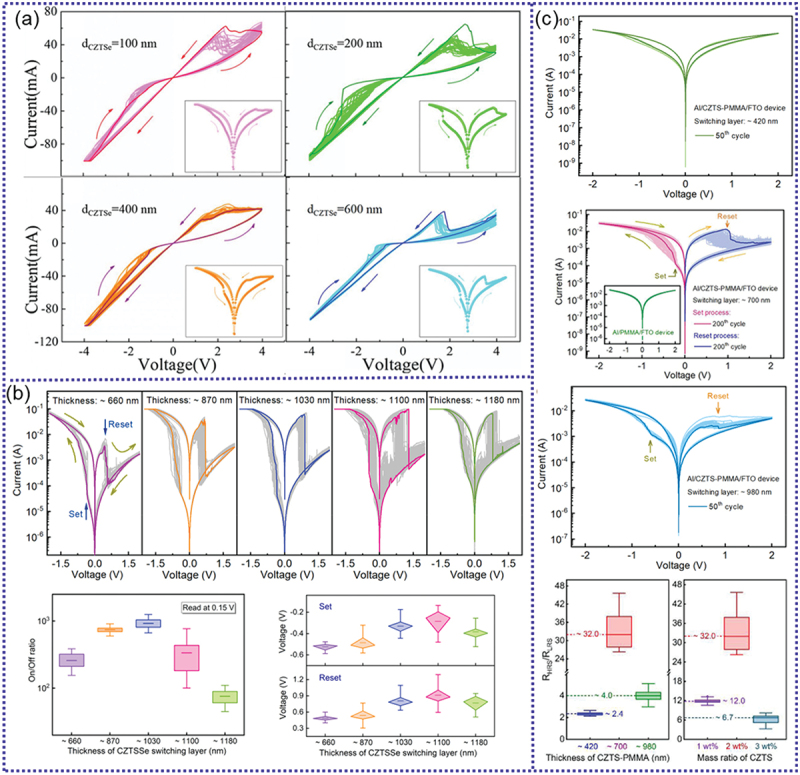
Thickness-dependent resistive switching performance. (a) AZO/CZTSe/FTO device with different CZTSe thicknesses (100–600 nm). Reproduced by permission from [58], Copyright 2018, Elsevier. (b) Ag/CZTSSe/CZTSSe/Mo device with different CZTSSe thicknesses (660–1180 nm). Reproduced by permission from [59] Copyright 2025, Elsevier. (c) Al/CZTS-PMMA/FTO/undefined/FTO device with different composite layer thicknesses (420–980 nm). Reproduced by permission from [60], Copyright 2024, AIP publishing.

Zheng et al. [58] fabricated an AZO/CZTSe/FTO structured memristor and systematically investigated the regulatory effect of the CZTSe resistive switching layer thickness (100, 200, 400, 600 nm) on the resistive switching performance of the device (Figure 2(a)). The results showed that when the thickness of the CZTSe functional layer was 200 nm, the device exhibited the most pronounced bipolar resistive switching hysteresis characteristics, achieving a maximum resistance ratio of the high-resistance state (HRS) to the low-resistance state (LRS) of ~6.855 at a read voltage of 0.175 V. In comparison, the sample with a thickness of 100 nm exhibited a significantly narrowed hysteresis loop, with a resistance ratio of only 5.463. As the thickness further increased to 400 nm and 600 nm, the resistive switching hysteresis characteristics continued to weaken, with the corresponding resistance ratios decreasing to 4.354 and 3.439, respectively. These thickness-dependent performance variations originate from the modulation effect of the Schottky barriers at the device interfaces. In the AZO/CZTSe/FTO device structure, Schottky contacts are formed at both the AZO/CZTSe interface and the CZTSe/FTO interface, and the synergistic effect of the two interfacial barriers dominates the internal carrier transport process. When the CZTSe layer is too thin (100 nm), the barrier heights at both interfaces decay too rapidly under forward bias, causing a sharp increase in the device current and potentially leading to barrier layer breakdown, ultimately weakening the hysteresis switching characteristics. When the CZTSe layer is too thick (≥400 nm), the width of the interfacial barrier region significantly increases, substantially reducing the carrier tunneling probability and thereby compressing the switching window. Only with a moderate thickness of 200 nm can an optimal match between the barrier height and width be achieved, balancing carrier injection and tunneling processes, thus yielding the optimal memory window. This study demonstrates that for memristors regulated by interfacial barriers, the resistive switching layer thickness exhibits a non-monotonic regulatory effect on switching performance, with a distinct optimal thickness range.

#### 2.1.2. CZTSSe switching films

Unlike the aforementioned devices dominated by interfacial barriers, the thickness optimization in the Ag/CZTSSe/Mo structure, where the conductive filament mechanism prevails, exhibits different trends. The research team of Cai et al. [59] fabricated CZTSSe resistive switching layers with varying thicknesses (660, 820, 1030, 1180 nm) in Ag/CZTSSe/Mo structured memristors (Figure 2(b)). Through systematic I‑V characterization, they elucidated the regulatory effect of thickness on the resistive switching performance, providing experimental support for thickness design in memristors governed by the conductive filament mechanism. The results show that as the CZTSSe film thickness increases from 660 nm to 1030 nm, the On/Off ratio (HRS/LRS) gradually improves, reaching a maximum value of approximately 1.25 × 10^3^ at 1030 nm. When the thickness further increases to 1180 nm, the On/Off ratio drops significantly. This exhibits a trend of first increasing and then decreasing. Regarding voltage characteristics, the Set voltage (V_Set_) is less affected by thickness within the range of −0.14 to −0.58 V. In contrast, the Reset voltage (V_Reset_) first increases and then decreases with increasing thickness. The V_Set_ and V_Reset_ distribution becomes broader, which is due to, in thicker CZTSSe films the formation position of conductive filaments is more random, resulting in higher and more widely distributed voltages required for filament rupture, in turn causing an increase in V_Set_ and V_Reset_.

Compared with the interfacial‑barrier‑dominated CZTSe device (optimal thickness 200 nm) [58], the conductive‑filament‑dominated CZTSSe device requires a thicker resistive switching layer (1030 nm) to achieve optimal performance. This is mainly because when the film thickness increases to the micrometer scale, the influence of the barrier with the electrode becomes negligible, and the resistive switching behavior is dominated by the formation and rupture of conductive filaments.

#### 2.1.3. CZTS-PMMA switching films

In the composite system of CZTS nanoparticles and PMMA polymer, the thickness of the resistive switching layer also affects device performance. Dong et al. [60] fixed the CZTS mass fraction at 2 wt.% and fabricated CZTS-PMMA composite resistive switching layers with different thicknesses (~420, 700, and 980 nm) by adjusting the number of spin-coating cycles (Figure 2(c)), systematically investigating the regulatory effect of thickness on the resistive switching performance of the devices. I‑V measurement results show that the device with a thickness of 700 nm exhibits the highest HRS/LRS ratio (average value ~32.0), whereas the On/Off ratio of the 420 nm and 980 nm devices are significantly lower. In this composite system, CZTS nanoparticles can act as electron trapping centers. This indicates the existence of an optimal thickness window in this composite system.

### 2.2. Element ratio regulation

In kesterite-structured CZTS, CZTSe, and CZTSSe materials, imbalances in the ratios of the constituent cations (Cu, Zn, Sn) can readily lead to issues such as the formation of secondary phases and abnormal defect concentrations, thereby altering the ion migration barrier and the distribution of trap states, ultimately affecting the resistive switching stability and power consumption levels of the devices [56,61]. Therefore, systematically investigating the regulatory effect of the elemental ratios in the resistive switching layer on memristor performance and clarifying the relationship between elemental proportions and key device performance parameters hold significant theory and practice importance for advancing the practical application of such memristors.

#### 2.2.1. Zn/Sn ratio

**Figure 3.**
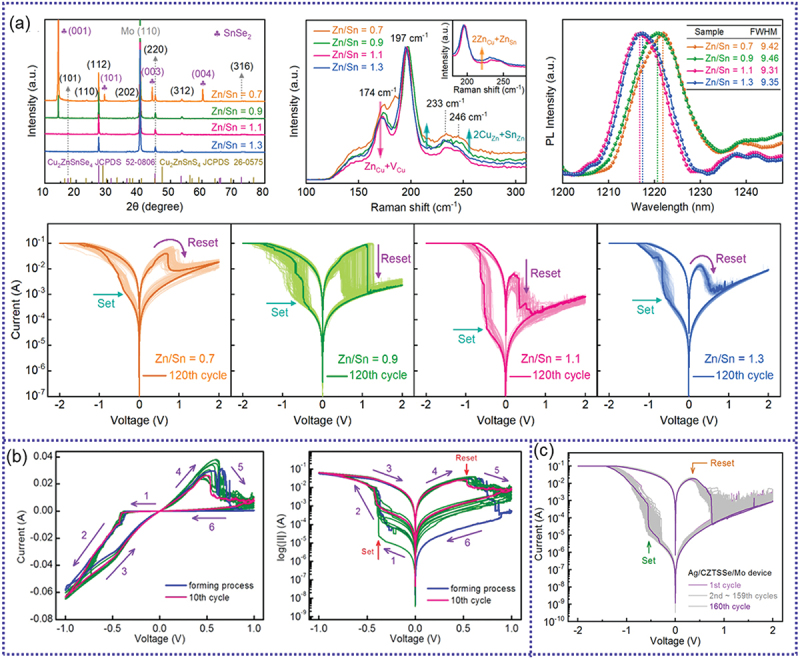
Effect of elemental ratios on memristor performance. (a) I‑V curves and On/Off ratio of Ag/CZTSSe/Mo devices with different Zn/Sn ratios (0.7–1.3). Reproduced by permission from [61], Copyright 2025, American Chemical society. (b) Coexistence of bipolar resistive switching and negative differential resistance in an Al/CZTSSe/Mo device with Cu‑poor CZTSSe (Cu/(Zn+sn) = 0.63). Reproduced by permission from [62], Copyright 2021, American Chemical society (c) bipolar resistive switching of the Ag/CZTSSe/Mo memristor with Cu/(Zn+Sn) =0.83. Reproduced by permission from [63], Copyright 2023, AIP publishing.

Because the phase-stability window of CZTSSe is very narrow, a balanced Zn/Sn ratio not only eliminates secondary phases such as SnSe_2_ but also leads to reduction in the concentrations of defect states (e.g. Cu_Zn_, Cu_Sn_) and defect clusters (e.g. Zn_Cu_ + V_Cu_), thereby significantly modulating the resistive switching behavior of the devices, making it one of the core parameters affecting the performance of CZTSSe memristors. To elucidate the regulatory mechanism of the Zn/Sn ratio, Yang et al. [61] designed and fabricated a series of CZTSSe thin films with Zn/Sn ratios of 0.7, 0.9, 1.1, and 1.3, and systematically investigated the influence of this ratio on memristor performance using an Ag/CZTSSe/Mo device structure (Figure 3(a)). Multiple characterization techniques (XRD, Raman, PL, FESEM) revealed that at a Zn/Sn ratio of 1.1, the CZTSSe film exhibited no SnSe_2_ secondary phases, the lowest defect concentration, a dense surface, and the largest grain size, thus yielding the best device performance. I‑V measurement results showed that all devices exhibited non‑volatile bipolar resistive switching behavior without the need for an initial electroforming process. The device with a Zn/Sn ratio of 1.1 displayed the lowest Set voltage (−0.38 ± 0.03 V) and Reset voltage (0.34 ± 0.05 V), which is beneficial for reducing power consumption. Notably, the On/Off ratio (HRS/LRS) varied dramatically with the Zn/Sn ratio: it increased from ~17 at a Zn/Sn ratio of 0.7 to about 63 at 0.9, reached approximately 1027 at 1.1, and then dropped back to about 149 at 1.3. The LRS was almost unaffected by the Zn/Sn ratio, whereas the HRS was highly sensitive to it. At low Zn/Sn ratios, the SnSe_2_ secondary phase provided more ion migration pathways, resulting in a low HRS. At a Zn/Sn ratio of 1.1, the pure, dense structure raised the migration barrier for Cu^+^ ions, significantly increasing the HRS.

#### 2.2.2. Cu content

In addition, variations in Cu content induce changes in the concentration of defects such as copper vacancies (V_Cu_) within the resistive switching layer, and also affect the distribution and migration of Cu ions, thereby jointly influencing the formation and rupture processes of conductive filaments. Dong et al. [62] fabricated an Al/CZTSSe/Mo device using a Cu‑poor CZTSSe thin film (Cu/(Zn+Sn) = 0.63) (Figure 3(b)). Despite the low copper content, the device exhibited stable coexistence of bipolar resistive switching and negative differential resistance, with an On/Off ratio of ~27.5. Yang et al. [63] fabricated an Ag/CZTSSe/Mo memristor using a CZTSSe film derived from a precursor solution with Cu/(Zn+Sn) = 0.83 (Figure 3(c)). The device exhibited stable bipolar resistive switching with a high On/Off ratio exceeding 10^4^, a low V_Set_ of −0.46 V and V_Reset_ of 0.35 V, and a retention time longer than 10^4^ s. Although the Cu content differs between the two devices, leading to distinct On/Off ratios and operating voltages, likely due to variations in V_Cu_ concentration, the resistive switching mechanism in both cases remains uniformly dominated by the formation and rupture of Cu conductive filaments.

### 2.3. Heterojunction and composite structures

Heterojunctions and composite structures represent effective approaches for modulating the electrical, optical, and memory performance of memristors. Through mechanisms such as interfacial energy level tuning, carrier transport optimization, and trap site modulation, they can effectively improve the switching characteristics, memory stability, and functional diversity of memristors [39,40,42]. Compared with single-layer memristors, heterojunctions can leverage the built-in electric field at the interface to achieve efficient carrier separation and regulation, while composite structures can combine the advantages of different components to synergistically optimize device performance. These strategies provide important insights for the design and fabrication of high-performance memristors and synaptic devices.

#### 2.3.1. CZTS/ZnO heterojunction

**Figure 4.**
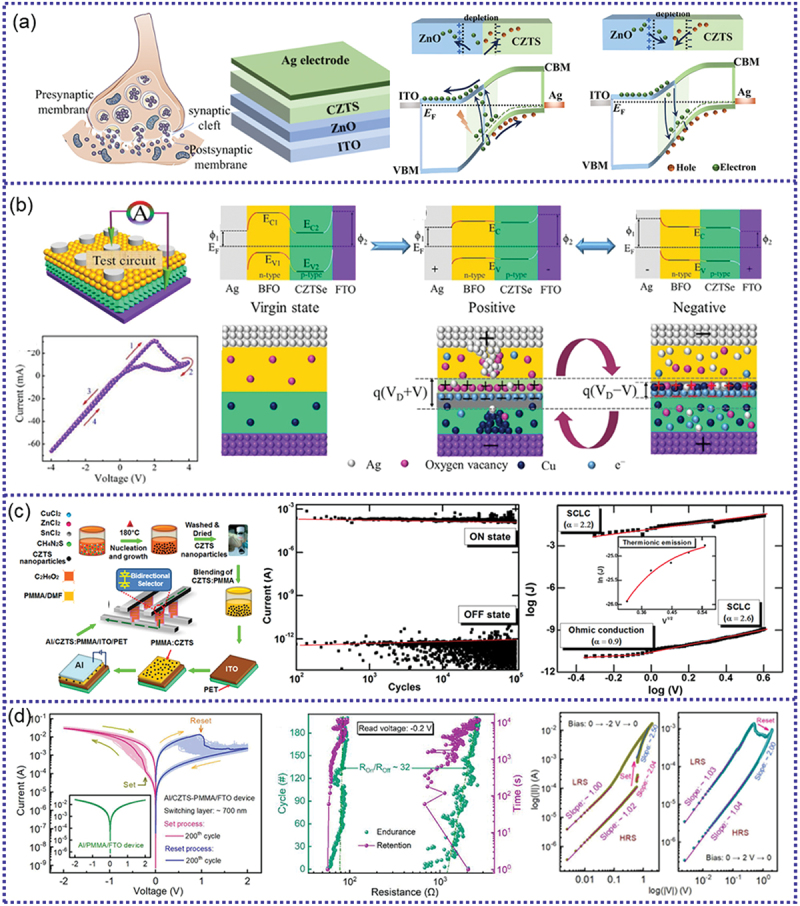
Heterojunction and composite structures. (a) Ag/CZTS/ZnO/ITO/CZTS/ZnO/ITO photoelectric synaptic device with type‑II heterojunction. Reproduced by permission from [64], Copyright 2024, Elsevier. (b) Ag/BFO/CZTSe/BFO/CZTSe/FTO heterojunction memristor showing multi‑field modulation. Reproduced by permission from [65]. (c) Al/CZTS/CZTS NPs:PMMA/ITO/PET organic bistable device with ultrahigh on/Off ratio. Reproduced by permission from [66], Copyright 2015, Elsevier. (d) Al/CZTS-PMMA/FTO/undefined/FTO device with optimized thickness for resistive switching. Reproduced by permission from [60], Copyright 2024, AIP publishing.

To achieve long-term memory retention in photoelectric synaptic devices, the team of Duan et al. [64] prepared CZTS thin films via a low-temperature annealing process and subsequently formed a type-II heterojunction with ZnO, successfully constructing an Ag/CZTS/ZnO/ITO structured photoelectric synaptic device (Figure 4(a)). Owing to the low-temperature annealing treatment, the CZTS film exhibits weak crystallinity, with a defect density as high as 1.42 × 10^16^ cm^−3^ and an optical bandgap of 1.63 eV. When the n-type ZnO and p-type CZTS form a heterojunction, a built-in electric field arises at the interface. Upon optical stimulation, this built-in field efficiently separates photogenerated electron–hole pairs: electrons drift toward the ZnO/ITO electrode, while holes migrate to the Ag electrode. Furthermore, defect levels located near the valence band of CZTS can trap a portion of the electrons. After the light stimulus is removed, the trapped electrons are gradually released and recombine with holes in the valence band, causing the EPSC to decay slowly and thereby achieving long-lasting memory retention.

#### 2.3.2. BFO/CZTSe heterojunction

Heterojunctions formed by multiferroic materials and chalcogenides offer unique advantages in the multi-field synergistic modulation of memristor performance. On this basis, Li and colleagues [65] focused on the heterojunction characteristics of BiFeO_3_ (BFO) and CZTSe, designing and investigating an Ag/BFO/CZTSe/FTO structured heterojunction memristor (Figure 4(b)). As a typical multiferroic material, BFO possesses both ferroelectricity and photoresponsive capability, while CZTSe is a p‑type chalcogenide. When the two form a heterojunction, band bending and a built‑in electric field are generated at the interface. I‑V measurements show bipolar resistive switching under different environmental conditions. The memory window varies significantly. At 1.025 V, the HRS/LRS ratio is ~2.1 (dark), ~4.5 (normal), ~6.0 (400 K heating), and ~22.4 (illumination). Combined mechanistic analysis reveals that under an external electric field, the Ag electrode ionizes to generate Ag^+^ ions, which migrate toward the interface. Under illumination, abundant photogenerated carriers are produced in both BFO and CZTSe, which accelerates the reduction of Ag^+^ to metallic Ag, forming Ag conductive filaments and switching the device to the LRS. Meanwhile, BFO undergoes polarization under the electric field, and its internal oxygen vacancies trap oxygen ions near the cathode, leading to the rupture of conductive pathways and the dissolution of Ag filaments, thereby returning the device to the HRS. This study fully demonstrates the excellent performance of the BFO/CZTSe heterojunction under the synergistic modulation of electric, optical, and thermal fields.

#### 2.3.3. CZTS-PMMA composite system

Embedding semiconductor nanoparticles into an insulating polymer matrix is an effective strategy to enhance the memory performance of organic bistable devices. Following this approach, Yun and colleagues [66] successfully fabricated organic bistable devices (OBDs) with an Al/CZTS NPs:PMMA/ITO/PET structure (Figure 4(c)) by incorporating CZTS nanoparticles into a PMMA insulating polymer matrix. I‑V measurements show that the device can be written and erased at +3.8 V and −2 V, respectively, achieving an On/Off current ratio as high as 1 × 10^9^. After 10^5^ cycling tests, the On/Off ratio of the device remains stable at the 10^9^ level, demonstrating excellent memory stability. The polycrystalline structure of the CZTS nanoparticles provides a large number of grain boundaries that serve as carrier trap sites, with the number of traps being far greater than that in mono‑element, binary, and ternary nanoparticles, which is a key reason for the large memory window obtained in this device.

To address the performance optimization of the CZTS-PMMA composite system, Dong et al. [60] fabricated Al/CZTS-PMMA/FTO devices and systematically optimized the thickness and mass fraction of the CZTS-PMMA composite layer (Figure 4(d)). With a CZTS mass fraction of 2 wt.% and a composite layer thickness of 700 nm, the device achieved optimal resistive switching performance, exhibiting an On/Off ratio of about 32 and a retention time exceeding 10^4^ s. The resistive switching behavior of the device follows the trap‑controlled space‑charge‑limited current (SCLC) mechanism. Compared with PMMA, CZTS possesses lower resistivity and a lower energy level, enabling it to act as an effective electron trapping center. The device successfully realized dual‑mode (electrical and optical) synaptic plasticity, encompassing functions such as EPSC, PPF, LTP/LTD, STDP, and decimal arithmetic operations. In comparison with the device fabricated by Yun et al. (which achieved an On/Off ratio as high as 10^9^) [66], the On/Off ratio of this device is relatively low (~32). However, its advantage lies in the realization of optoelectronic dual‑mode synaptic functionality, making it more suitable for neuromorphic computing applications rather than purely digital memory storage.

### 2.4. Electrode engineering

**Figure 5.**
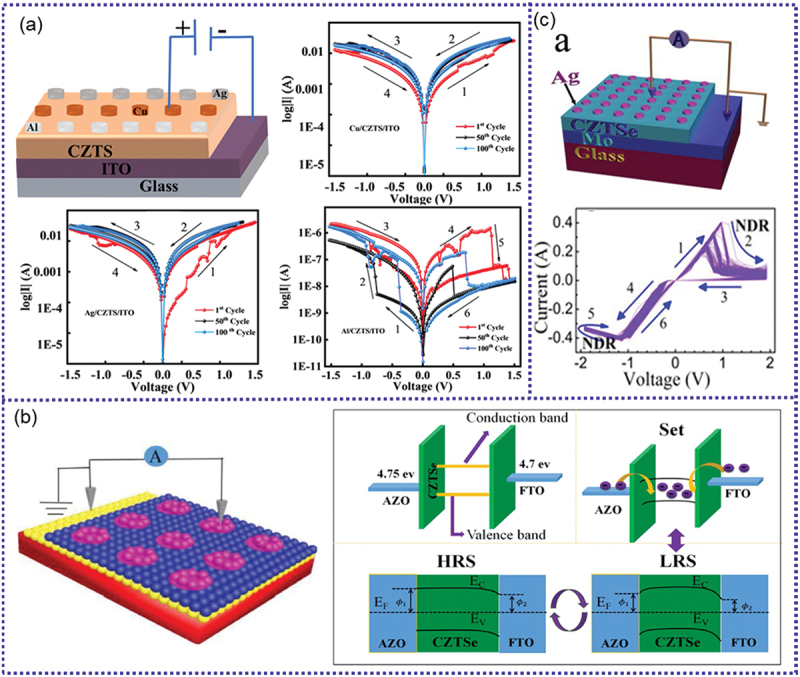
Electrode engineering effects. (a) Comparison of Cu, Ag, and Al top electrodes on Cu‑rich CZTS memristors (M/CZTS/ITO). Reproduced by permission from [67], Copyright 2023, John Wiley and Sons. (b) AZO/CZTSe/FTO memristor with transparent conductive oxide electrodes. Reproduced by permission from [58], Copyright 2018, Elsevier. (c) Coexistence of resistive switching and NDR in an Ag/CZTSe/Mo device. Reproduced by permission from [68], Copyright 2018, Royal Society of Chemistry.

The choice of top electrode material not only determines the Schottky barrier height at the metal/semiconductor interface but also directly affects the formation mechanism of conductive filaments [42,47,50]. Therefore, electrode engineering serves as an effective means to modulate the switching type, On/Off ratio, stability, and power consumption. To investigate the influence of different metal top electrodes on the performance of CZTS-based memristors, Yadav et al. [67] systematically compared the effects of three top electrodes, namely Cu, Ag, and Al, on Cu‑rich CZTS film memristors (M/CZTS/ITO) (Figure 5(a)). I‑V measurements revealed that devices with Cu and Ag electrodes exhibited analog bipolar resistive switching, with a smooth variation of current as a function of voltage, whereas the device with an Al electrode showed digital bipolar resistive switching, characterized by abrupt current changes at the Set/Reset voltages. In terms of On/Off ratio, the Al‑electrode device exhibited the highest value (HRS/LRS ~32.2), followed by the Cu‑electrode device (~4) and the Ag‑electrode device (~2). The analysis suggests that for Cu and Ag electrodes, the electrodes themselves ionize under an electric field to generate Cu^+^/Ag^+^ ions, which together with the intrinsic Cu^+^ ions from CZTS participate in the formation and rupture of conductive filaments. The gradual nature of ion migration leads to analog switching. In contrast, Al is an inert metal that does not provide additional ions and relies solely on the intrinsic Cu^+^ from CZTS. As a result, filament formation exhibits a more threshold‑like behavior, yielding digital switching, and the absence of interference from electrode ions results in a higher On/Off ratio.

In addition to metal electrodes, transparent conductive oxide electrodes have also been employed in the study of CZTS-based memristors and influenced the device property. Zheng et al. [58] constructed a device of FTO/CZTSe/AZO, where the bottom electrode is fluorine-doped tin oxide (FTO), the top electrode is aluminum-doped zinc oxide (AZO) (Figure 5(b)). Experimental results show that the nonvolatile resistive switching memory effect in this device does not originate from the formation of conductive filaments, but is attributed to the Schottky barriers formed at the two interfaces of FTO/CZTSe and CZTSe/AZO. Due to the different work functions of FTO and AZO, Schottky junctions are formed when they contact CZTSe. Under an applied bias, the width of the interfacial barrier is modulated, thereby controlling the carrier tunneling behavior: when a positive voltage is applied to the top electrode AZO, the Schottky barrier at the CZTSe/AZO interface is lowered, switching the device to LRS; under reverse bias, the barrier is raised, returning the device to HRS. This work reveals the key role of transparent electrodes FTO and AZO in forming interfacial barriers and dominating carrier-tunneling-type resistive switching, providing a non-filamentary physical model for memristors.

To elucidate the regulatory mechanism of electrode combinations on the special electrical phenomena in memristors, Guo et al. [68] investigated the influence of Ag and Mo electrodes on the coexistence of resistive switching and negative differential resistance (NDR) in an Ag/CZTSe/Mo structure (Figure 5(c)). The device exhibited bipolar resistive switching performance at room temperature, with an HRS/LRS ratio of ~ 215 and a retention time of about 10^4^ s. NDR effects were observed in both forward and reverse scans. Specifically, when the applied voltage increased to a certain threshold, the device current no longer rose with increasing voltage but instead exhibited a marked decrease. Mechanistic analysis reveals that the CZTSe film used in the Ag/CZTSe/Mo device is slightly Cu‑rich. Under an external electric field, Cu^2+^ ions in the active layer, which possess high mobility and reactivity, migrate toward the cathode and are reduced to form Cu conductive filaments. The Ag top electrode and Mo bottom electrode primarily serve as conductive pathways and contribute to the formation of interfacial Schottky barriers rather than directly participating in filament formation. When the bias voltage increases to a sufficiently high level, local Joule heating effects cause the rupturing of Cu conductive filaments, switching the device from LRS to HRS. Meanwhile, V_Cu_ accumulate at the Ag/CZTSe and CZTSe/Mo interfaces, raising the Schottky barriers and hindering carrier injection, thereby giving rise to NDR. Furthermore, the Mo bottom electrode forms a Schottky contact with CZTSe, creating a back‑to‑back double junction with Ag/CZTSe, and the accumulation of V_Cu_ at these interfaces is considered the main cause of the NDR effect.

### 2.5. Environmental and operating mode tuning

**Figure 6.**
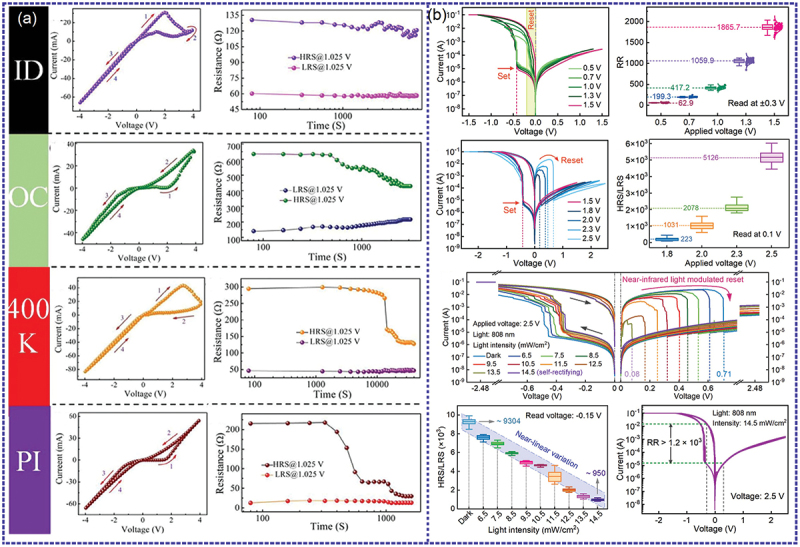
Influence of testing environment and operating mode. (a) Resistive switching behavior of Ag/BFO/CZTSe/FTO under different conditions (dark, normal, 400 K heating, illumination). Reproduced by permission from [65]. (b) Mode switching between self‑rectifying and bipolar resistive switching in an Ag/CZTSSe/Mo device controlled by voltage and light intensity. Reproduced by permission from [69], Copyright 2025, John Wiley and Sons.

In practical applications, memristors often need to adapt to complex and variable operating environments; therefore, investigating the influence of environmental factors (such as temperature, illumination, etc.) and operating modes on device performance is of great significance. Li et al. [65] systematically studied the effects of different testing environments (dark, normal conditions, 400 K heating, and illumination) on the resistive switching behavior in an Ag/BFO/CZTSe/FTO structure (Figure 6(a)). The results show that the HRS/LRS resistance ratio of the device varies significantly under different environmental conditions: approximately 5 in the dark, about 45 under normal conditions, roughly 25 under heating at 400 K, and as high as approximately 4500 under illumination. This finding indicates that illumination can significantly enhance the switching window of the device. Furthermore, Yang et al. [69] achieved reversible switching between two operating modes, self‑rectifying and bipolar resistive switching, in a single Ag/CZTSSe/Mo structure (Figure 6(b)). Under low‑voltage scanning conditions (<1.5 V), the device exhibits self‑rectifying characteristics with a rectification ratio exceeding 10^3^. When the applied voltage exceeds 1.5 V, the device switches to bipolar resistive switching mode, with an On/Off ratio that is bias‑tunable within the range of 2.2 × 10^2^ to 5.1 × 10^3^. In addition, the device also exhibits pronounced photo‑tunable characteristics, further expanding the dimensionalities of operating mode control. Under 808 nm near‑infrared light irradiation, the Reset voltage of the device gradually decreases from 0.52 V in the dark to 0.06 V at a light intensity of 13.5 mW/cm^2^. When the light intensity exceeds 13.5 mW/cm^2^, the device switches from bipolar switching mode to self‑rectifying mode, achieving a rectification ratio of 1.2 × 10^3^. This light‑intensity‑driven mode switching provides a new route for optoelectronic synergy modulation.

## 3. Emulation of synaptic plasticity

### 3.1. Optically modulated synaptic plasticity

**Figure 7.**
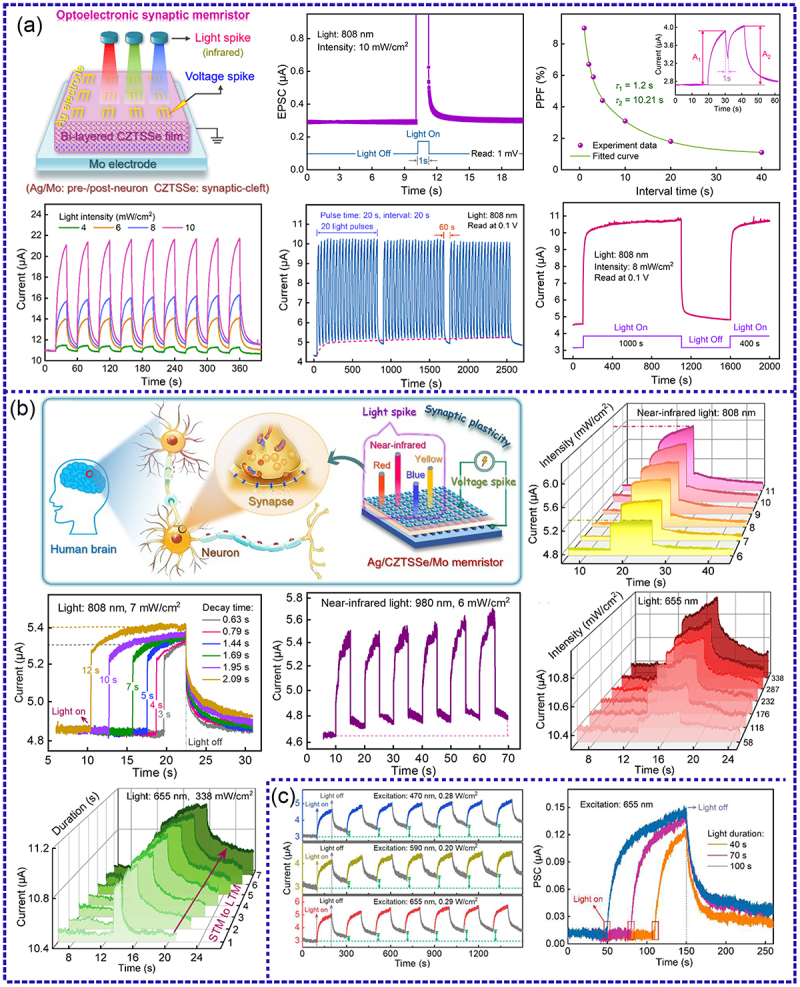
Optically modulated synaptic plasticity. (a) EPSC, PPF, and long‑term memory under 808 nm light pulses in an Ag/CZTSSe/Mo device. Reproduced by permission from [59], Copyright 2025, Elsevier. (b) Light intensity and pulse duration effects on short‑term to long‑term memory transition. Reproduced by permission from [69], Copyright 2025, John Wiley and Sons. (c) Multi‑wavelength visible light (470, 590, 655 nm) modulation in an Al/CZTS-PMMA/FTO device. Reproduced by permission from [60], Copyright 2024, AIP Publishing.

Synaptic plasticity, as the core foundation of learning, memory, and information processing in neural networks, makes the investigation of its regulatory mechanisms critically important for the development of novel neuromorphic devices [8,20]. Optical modulation, with its unique advantages such as fast response speed, high regulation precision, clean and contactless operation, and the ability to achieve precise multi‑wavelength control, offers a new and efficient technological pathway for synaptic plasticity research [25,38,41]. Kesterite‑based memristors, owing to their excellent optoelectronic properties and synaptic simulation potential, have become ideal platforms for optically modulated synaptic plasticity studies. On this basis, relevant research has focused on kesterite‑based memristors to explore optical regulation mechanisms and synaptic behavior simulation. Among these efforts, Lan et al. [59] conducted systematic research on Ag/CZTSSe/Mo memristor devices (Figure 7(a)). First, the basic synaptic function was tested using single optical pulse stimulation. A single 808 nm light pulse induced an excitatory postsynaptic current (EPSC), which rapidly increased when the light pulse was turned on and gradually decayed after it was turned off, successfully mimicking the excitatory transmission process of biological synapses. Subsequently, to explore the effect of pulse parameter on synaptic behavior, by varying the time interval between two consecutive light pulses, the variation of the paired‑pulse facilitation (PPF) index with increasing interval was clearly observed. Meanwhile, to analyze the regulatory effect of light intensity on memory effects, the responses to cyclic pulse stimulation under different light intensities (4, 6, 8, 10 mW/cm^2^) were measured. The results showed that higher light intensity led to larger peak photocurrent and residual current after the light was turned off, indicating that stronger light stimulation induces a more persistent memory effect. Furthermore, the current responses under three sequences of 20 light pulses each exhibited good light response, and after each sequence, the residual current after decay showed a gradual increasing trend, successfully simulating the reinforcement of memory through repeated learning. Notably, the device also exhibited long‑term memory behavior under 808 nm light stimulation: when the light stimulation duration was extended to 1000 s, the photocurrent gradually increased and stabilized; after the light was turned off for 500 s, reapplying light stimulation for 400 s restored the photocurrent to the same level as before, fully demonstrating memory consolidation and rapid recovery capability.

Yang et al. [69] further investigated the modulatory effect of 808 nm light pulses (Figure 7(b)), obtaining results partially consistent with those of Lan et al. [59], while additionally revealing the influence of light intensity and pulse duration on the type of synaptic memory. When the light intensity increased from 6 mW/cm^2^ to 11 mW/cm^2^, the peak photocurrent increased accordingly. Under low light intensity, the current rapidly decayed to baseline, corresponding to simulated short‑term memory (STM); under high light intensity, a higher residual current was maintained, corresponding to simulated long‑term memory (LTM). In addition to light intensity, extending the light pulse width (3–12 s) also significantly enhanced the postsynaptic current and slowed its decay rate, increasing the decay time constant from 0.63 s to 2.09 s. Furthermore, under 980 nm near‑infrared light stimulation, the device successfully mimicked the learning‑memory consolidation rules of biological synapses.

It is worth noting that optical modulation is not limited to the near‑infrared band; the visible light band can also effectively achieve synaptic plasticity modulation. Yang et al. [69] further studied the regulatory effects of three visible wavelengths (470, 590, 655 nm) on the synaptic behavior of the device and found that by adjusting light intensity or illumination time, the transition from STM to LTM could be successfully achieved. Weak or short‑duration stimulation only induced transient photocurrent, which rapidly decayed after stimulus removal, corresponding to STM. Strong or long‑duration stimulation produced a higher steady‑state residual current with a significantly reduced decay rate, corresponding to LTM.

In addition to the above devices, Dong et al. [60] studied the synaptic modulation effect of multi‑wavelength visible lights (470, 590, 655 nm) in an Al/CZTS‑PMMA/FTO device (Figure 7(c)). Upon each light illumination, the photocurrent rapidly increased, and after the light was turned off, it slowly decayed. Moreover, after multiple cycles of stimulation, the residual current after decay accumulated progressively. This characteristic successfully simulated the dynamic process of ‘learning‑forgetting‑consolidation’ in biological synapses, further enriching the research content of visible‑light‑modulated synaptic plasticity.

### 3.2. Electrically modulated synaptic plasticity

Compared with optical modulation, electrical pulse modulation offers advantages such as precise signal timing, compatibility with existing complementary metal-oxide-semiconductor (CMOS) technology, and ease of large‑scale array programming, making it the most common and direct approach for simulating dynamic synaptic behaviors. Memristors, with their conductance continuously adjustable according to the history of pulse stimulation, can map the weight changes of biological synapses and have thus become core devices for electrically modulated synaptic plasticity [1,7]. By designing pulse amplitude, width, interval, and spike timing, memristors can effectively emulate key synaptic functions such as EPSC, PPF, long‑term potentiation/depression (LTP/LTD), spike‑timing‑dependent plasticity (STDP), and ‘learning‑relearning’ [38,39,44]. Kesterite‑based memristors, owing to their tunable composition, environmental friendliness, and high nonlinearity of conductance modulation, have achieved a series of advances in the field of electrical pulse synaptic simulation.

**Figure 8.**
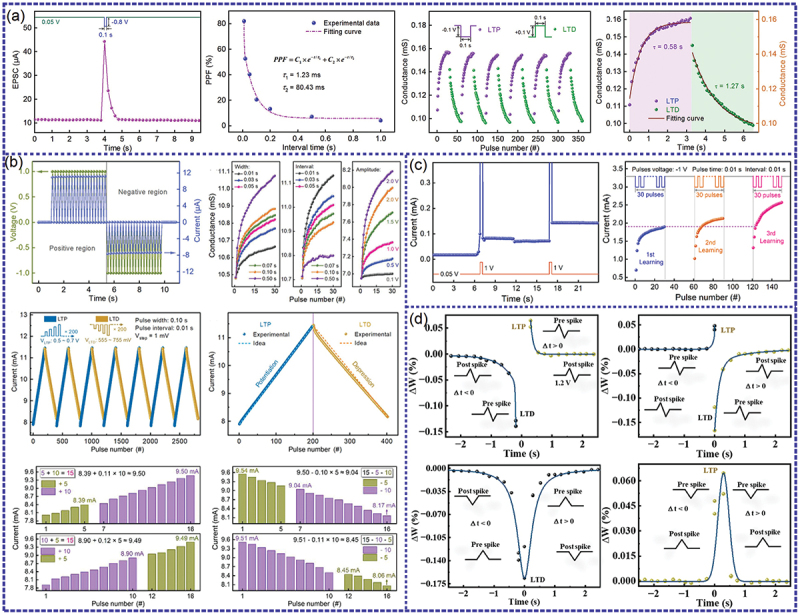
Electrically modulated synaptic plasticity. (a) EPSC, PPF, and LTP/LTD in a Ag/CZTSSe/Mo memristor. Reproduced by permission from [63], Copyright 2023, AIP Publishing. (b) Pulse‑width/interval/amplitude modulation and decimal arithmetic operations in an Al/CZTS-PMMA/FTO device. Reproduced by permission from [60], Copyright 2024, AIP Publishing. (c) Learning‑relearning behavior in an Ag/CZTSSe/Mo memristor. Reproduced by permission from [59], Copyright 2025, Elsevier. (d) STDP learning rules (Hebbian and anti‑Hebbian) in a Cu/CZTS/ITO device. Reproduced by permission from [70].

In the study of CZTSSe-based memristors, Yang et al. [63] successfully simulated a variety of key synaptic behaviors using electrical pulse stimulation (Figure 8(a)). When a negative voltage pulse with specific parameters was applied to the top electrode, the device generated an EPSC response, characterized by an abrupt increase in current under the pulse followed by a decay to pre‑stimulation levels, thereby mimicking short‑term synaptic plasticity. Significant PPF features were observed: under pulse stimulation of identical amplitude and duration, the PPF index was higher at short intervals, and the decay time was consistent with results from biological synapse tests. Under continuous identical pulse stimulation, the conductance of the device could be finely modulated by negative pulses (potentiation) and positive pulses (depression), showing a sustained increase or decrease with consecutive pulse trains and gradually approaching saturation. This demonstrates excellent LTP/LTD simulation capability, exhibiting exponential changes during both potentiation and depression processes. These results indicate that the CZTSSe-based memristor possesses outstanding synaptic function simulation capabilities, closely matching the characteristics of biological synapses.

The Al/CZTS-PMMA/FTO device reported by Li’s group [60] can achieve continuous fine-tuning of current through positive and negative triangular pulses, mimicking the weight modulation of biological synapses (Figure 8(b)). Its conductance can also be continuously regulated by pulse width, interval, and amplitude, with different pulse parameters inducing corresponding increases or decreases in conductance. Furthermore, under staircase pulse stimulation, the device exhibits symmetric LTP and LTD behaviors with multi‑cycle programmable modulation, where the current varies linearly with the number of pulses. By virtue of its linearly tunable current characteristics, the device successfully realizes precise decimal arithmetic calculations obeying the commutative laws of addition and subtraction. The validity of the commutative laws of addition and subtraction was verified through different sequences of staircase pulse stimulation, further demonstrating the excellent synaptic modulation capability and computational potential of the device.

In another study, Lan et al. [59] investigated the EPSC response of Ag/CZTSSe/Mo memristors under continuous electrical pulse stimulation and found that the steady‑state current induced by subsequent pulses was higher than that induced by the previous pulse, indicating a short‑term plasticity accumulation effect in the device (Figure 8(c)). More importantly, the device successfully mimicked the ‘learning‑relearning’ behavior: as the number of learning sessions increased, the number of pulses required to reach the same current level decreased significantly, and eventually a higher current value could be restored, vividly reflecting the cognitive rule of improved learning efficiency after knowledge consolidation. Furthermore, Yadav et al. [70] studied the STDP behavior of Cu/CZTS/ITO resistive random-access memory (RRAM) devices (Figure 8(d)). The device achieved both asymmetric and symmetric Hebbian and anti‑Hebbian learning rules, with synaptic weight exhibiting LTP or LTD changes as a function of the spike‑timing difference (Δt) between pre‑ and post‑synaptic spikes. The time constants and conductance changes under different learning rules were obtained by fitting, and the pulse timing interval had a significant effect on the synaptic weight change: the shorter the interval, the more pronounced the conductance change, and the conductance difference gradually approached zero as the interval increased.

## 4. Advanced neuromorphic function applications

Kesterite-based optoelectronic synaptic memristors, leveraging their dual electrical/optical field-modulated resistive switching characteristics, self-rectifying capability, and rich defect-state dynamics, have broken through the limitations of traditional storage and computing, expanding into a variety of highly promising application scenarios such as information security, neuromorphic computing, and visual perception.

### 4.1. Information encryption and secure communication

**Figure 9.**
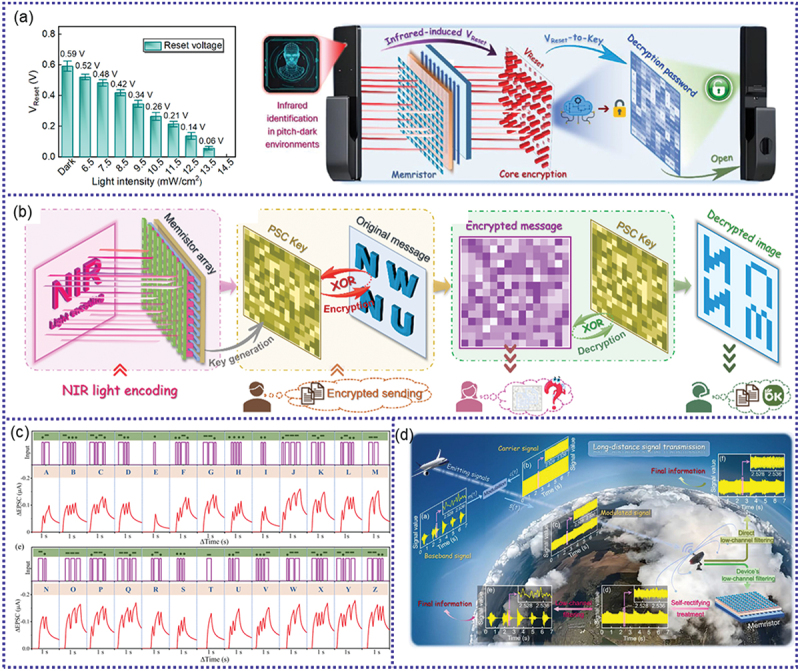
Information encryption and secure communication. (a) Dynamic physical unclonable function (PUF) encryption based on NIR‑tunable Reset voltage in an Ag/CZTSSe/Mo device. Reproduced by permission from [69], Copyright 2025, John Wiley and Sons. (b) PUF‑based encryption key for image XOR operation. Reproduced by permission from [61], Copyright 2025, American Chemical society. (c) Morse code encryption using pulse-width dependent plasticity in Ag/CZTS/ZnO/ITO device, encoding letters A-Z. Reproduced by permission from [64], Copyright 2024, Elsevier. (d) Half‑wave rectification and signal recovery using the self‑rectifying property for long‑distance communication. Reproduced by permission from [69], Copyright 2025, John Wiley and Sons.

Leveraging the unique physical characteristics of kesterite‑based memristors, they exhibit significant advantages in the fields of information encryption and secure communication. The core concept utilizes the near‑infrared (NIR) tunable Reset voltage, multilevel conductance states, and self‑rectifying properties of CZTSSe devices to achieve hardware‑level encryption protection and efficient signal demodulation. Yang et al. [69] discovered that in an Ag/CZTSSe/Mo device, the Reset voltage can be linearly modulated by 808 nm NIR light intensity (Figure 9(a)). Based on this property, they proposed a dynamic physical unclonable function (PUF) encryption scheme. Biometric features or target morphologies acquired via infrared recognition are mapped to NIR light of different intensities, which are then illuminated onto a memristor array. Each cell outputs a unique Reset voltage value according to the received light intensity, thereby generating a dynamic voltage matrix cipher bound to the target. The system compares the real‑time generated cipher with a pre‑stored key; when the similarity exceeds a threshold, unlocking is granted. This method effectively resists traditional static password and biometric spoofing attacks, offering a new approach for hardware security. Also utilizing NIR‑modulated multilevel conductance, Yang et al. [61] further constructed a PUF‑based encryption key (Figure 9(b)). During encryption, the original image pixel matrix is subjected to an exclusive OR (XOR) operation with the key, outputting statistically uniform random noise that completely masks the original features. Only a receiving end holding the same physical key can perform XOR again to restore the original image. This hardware‑algorithm collaborative encryption strategy significantly enhances the security of information transmission. Meanwhile, the modulation of light pulse width provides an alternative approach for information encryption. Mao et al. [64] in a CZTS/ZnO heterojunction device, exploited pulse-width-dependent plasticity to emulate Morse code communication: a 50 ms light pulse represented a dot, and a 150 ms light pulse represented a dash (Figure 9(c)). By combining light pulse sequences of different widths, they achieved encoding and transmission of the 26 letters of the alphabet and successfully demonstrated encryption of the word ‘ECNU’. This hardware‑algorithm collaborative encryption strategy significantly enhances the security of information transmission.

In addition to information encryption, this class of devices also holds important applications in long-distance communication scenarios. In long‑distance transmission contexts such as aeronautical communications, Yang et al. [69] utilized the self-rectifying property of the Ag/CZTSSe/Mo device to perform half-wave rectification on an amplitude‑modulated signal with a 10 kHz carrier, preserving the baseband envelope, and then accurately recovered the original low‑frequency signal after low‑pass filtering (Figure 9(d)). This scheme provides a new approach for long‑distance high‑precision data transmission.

### 4.2. Neuromorphic computing and pattern recognition

**Figure 10.**
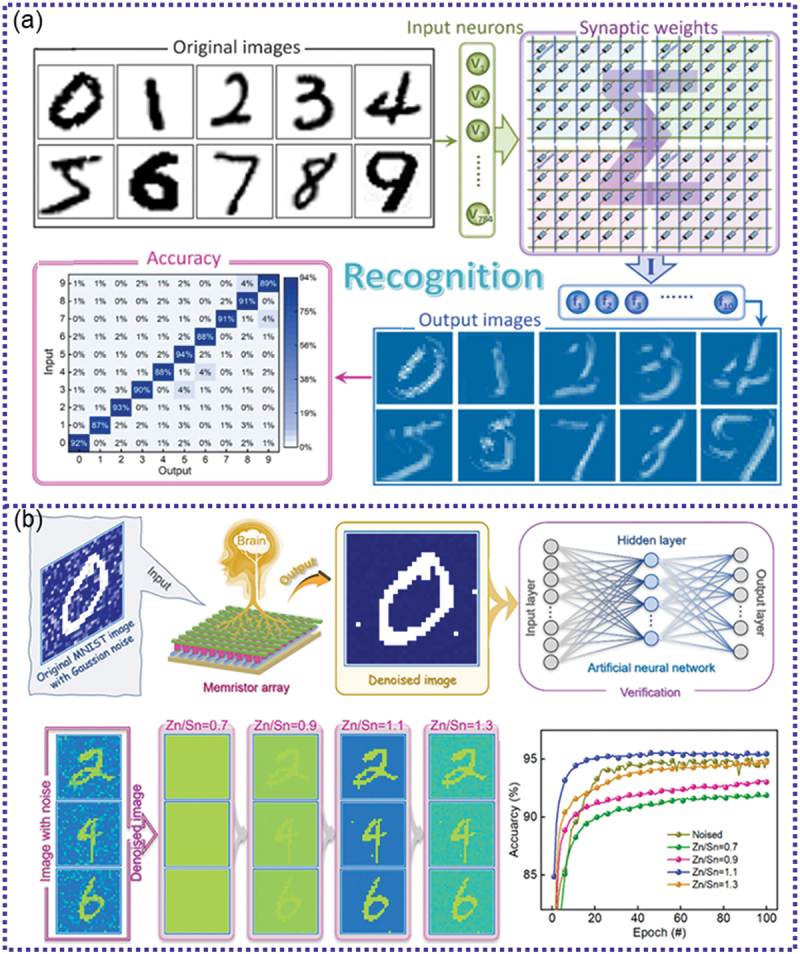
Neuromorphic computing and pattern recognition. (a) Three‑layer fully connected neural network (784‑100‑10) for MNIST handwritten digit recognition using an Al/CZTS-PMMA/FTO device, achieving 91.3% accuracy. Reproduced by permission from [60], Copyright 2024, AIP Publishing. (b) Gaussian noise image denoising strategy based on On/Off ratio modulation in CZTSSe memristors, with recognition rate up to 95.11%. Reproduced by permission from [61], Copyright 2025, American Chemical Society.

Neuromorphic computing and pattern recognition represent another application domain for kesterite‑based optoelectronic synaptic memristors. The core logic lies in leveraging the linear, symmetric, and multilevel conductance modulation capability of memristors to construct artificial neural networks, thereby efficiently executing numerical operations and image recognition tasks. Dong et al. [60] achieved linear modulation of LTP and LTD in an Al/CZTS‑PMMA/FTO device (Figure 10(a)). Based on this characteristic, they constructed a three‑layer fully connected neural network with an input layer (784 neurons), a hidden layer (100 neurons), and an output layer (10 neurons) for MNIST handwritten digit recognition. After 100 training epochs, the recognition accuracy of this hardware neural network reached 91.3%, which is very close to the 92.2% achieved by pure software simulation. The confusion matrix showed that the recognition accuracy for individual digits ranged between 87% and 94%, fully validating the feasibility of this device for neuromorphic computing.

Yang et al. [61] found that adjusting the Zn/Sn ratio of CZTSSe memristors can effectively modulate the device On/Off ratio. Leveraging this characteristic, they proposed a Gaussian noise image denoising strategy based on memristors (Figure 10(b)). High-intensity and low‑intensity pixels were mapped to the HRS and LRS of the device, respectively, thereby effectively distinguishing noise from image structural information. The denoised images were then input into a three‑layer neural network for MNIST handwritten digit recognition. The results showed that as the On/Off ratio increased, the denoising effect and recognition accuracy were significantly improved, reaching a maximum recognition rate of 95.11% at a Zn/Sn ratio of 1.1, verifying the advantages of high‑switching‑ratio devices in image detail enhancement and feature extraction.

### 4.3. Visual perception and associative learning

**Figure 11.**
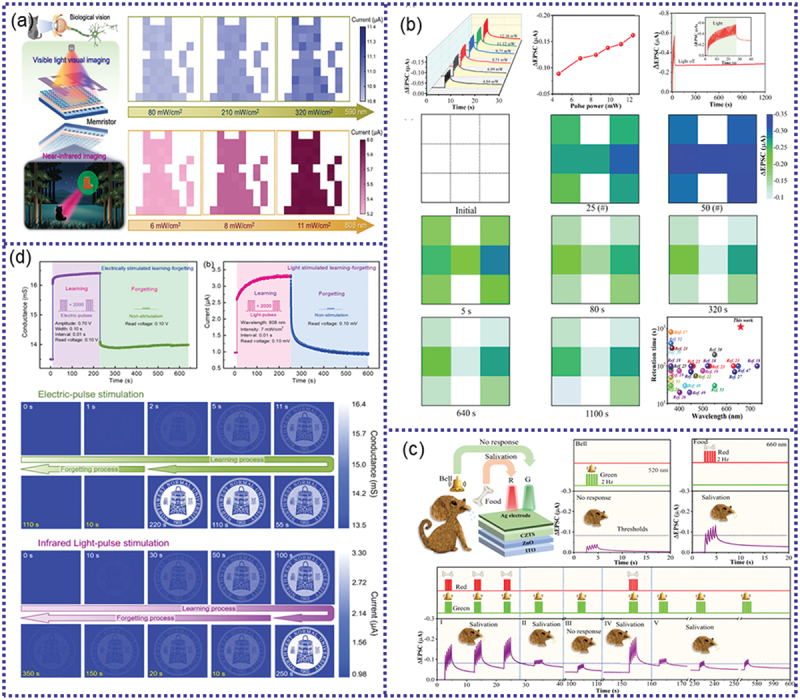
Visual perception and associative learning. (a) 10 × 10 Ag/CZTSSe/Mo array for visual perception, night vision imaging, and distance‑based image generation. Reproduced by permission from [69], Copyright 2025, John Wiley and Sons. (b) Long‑term image retention in an Ag/CZTS/ZnO/ITO device. (c) Pavlovian conditioning experiment using 520 nm (bell) and 660 nm (food) light pulses. Reproduced by permission from [64], Copyright 2024, Elsevier. (d) Learning‑forgetting process following the Ebbinghaus curve under NIR light stimulation, showing long‑lasting memory. Reproduced by permission from [60], Copyright 2024, AIP Publishing.

Leveraging the broadband photoresponse (covering the visible to near-infrared wavelength range) and defect-trapping-induced persistent photoconductivity of kesterite-based materials, kesterite-based memristors can efficiently simulate the perception, memory, and associative learning functions of biological visual systems, providing significant support for the construction of brain-inspired vision systems. Yang et al. successfully simulated biological visual perception and memory functions using a 10 × 10 Ag/CZTSSe/Mo memristor array (Figure 11(a)). Under 590 nm visible light stimulation, the photoconductance of the device increases with light intensity, and the chromaticity variation in the heatmap intuitively reflects the light-intensity-dependent memory consolidation process. In low-light environments, the array performs detection using 808 nm near-infrared light and generates high-fidelity images based on target distance, thereby simulating night vision imaging capabilities.

The persistence of image memory is a key metric for evaluating the performance of visual perception devices. Most optoelectronic synaptic devices exhibit an image retention time of less than 100 s after the removal of optical stimulation. In contrast, the Ag/CZTS/ZnO/ITO device fabricated by Mao et al. [64] demonstrates excellent performance in this regard (Figure 11(b)). Using 660 nm light pulses to encode an ‘H’ pattern on a 3 × 3 pixels array, the image intensity gradually increases with the number of pulses. After the removal of optical stimulation, the pattern remains discernible for 1150 s, with the residual current retaining 49.7% of its initial peak value. This represents a significant advantage over most similar devices, highlighting the non‑volatile image memory capability enabled by the defect‑trapping mechanism. Furthermore, a Pavlovian conditioning experiment was successfully simulated using 520 nm (green light, simulating a bell) and 660 nm (red light, simulating food) light pulses (Figure 11(c)): green light alone elicited no response, while red light alone induced an unconditioned reflex. After three sessions of paired green‑red light stimulation, green light alone produced a conditioned response exceeding the threshold. The conditioned response weakened over time but was restored and strengthened upon re‑stimulation with the paired stimuli. This simulation lays the foundation for the application of optoelectronic synaptic devices in brain‑inspired associative learning.

In addition to visual perception and associative learning, this class of devices can also simulate the ‘learning‑forgetting’ process of the biological brain. Dong et al. successfully mimicked the ‘learning‑forgetting’ process conforming to the Ebbinghaus forgetting curve by exploiting the cumulative enhancement and spontaneous decay characteristics of the device conductance under repeated electrical or optical pulse stimulation (Figure 11(d)). Experiments show that the Al/CZTS‑PMMA/FTO memristor exhibits significantly different learning‑forgetting behaviors under electrical pulse and near‑infrared (NIR) light pulse stimulation. Under electrical pulse training, the simulated school badge pattern required 220 s to become clearly visible, but the image disappeared rapidly after stimulus removal, failing to effectively replicate the forgetting process. In contrast, under 808 nm NIR light stimulation, the device not only achieved a dynamic process resembling biological ‘learning‑memory‑forgetting’, but also maintained the memory for more than 350 s, a behavior that precisely matches the ‘learning‑memory‑forgetting’ rule of the biological brain. The light‑induced persistent photoconductivity effect enables long‑term retention of memory traces. This visual simulation not only validates the device’s ability to reproduce the biological learning‑forgetting curve, but also highlights the significant advantage of optical stimulation over electrical stimulation in achieving long‑term memory, providing experimental evidence for the construction of brain‑inspired vision systems with persistent memory capability.

## 5. Conclusion and perspectives

This article provides a systematic overview of the research progress on kesterite‑based memristors as emerging optoelectronic neuromorphic devices. Benefiting from their tunable bandgap and excellent broadband light absorption capability, kesterite‑based materials offer a promising new technological pathway for developing high‑performance optoelectronic synaptic memristors. Studies have shown that by optimizing resistive switching layer thickness, elemental ratios, heterojunctions, electrode materials, adjusting sweep voltage parameters, and introducing near‑infrared light synergistic stimulation, the resistive switching performance can be effectively modulated, including On/Off ratio, operating voltage, and self‑rectifying characteristics of the devices. More importantly, under electrical pulse and broadband light stimulation, such devices have successfully emulated various biological synaptic functions ranging from short‑term plasticity to long‑term plasticity, and have exhibited advanced cognitive behaviors driven by optoelectronic synergy, such as the ‘learning‑forgetting‑relearning’ process and classical conditioning. Leveraging their excellent physical properties, kesterite‑based memristors have demonstrated significant potential in multiple neuromorphic application scenarios: in information encryption and secure communication, they have achieved dynamic key encryption based on physical unclonable functions (PUF), image XOR encryption, and long‑distance analog signal demodulation; in neuromorphic computing and pattern recognition, they have been successfully used for MNIST handwritten digit recognition and image Gaussian noise denoising based on On/Off ratio modulation; in visual perception and associative learning, they have realized near‑infrared night vision imaging, long-time visual image retention, and Pavlovian conditioning associative learning. These outcomes highlight the important value of kesterite‑based memristors in realizing integrated perception‑storage‑computation intelligent systems.

Currently, the development of kesterite‑based memristors still faces considerable challenges and innovative opportunities. First, by modulating the distribution of vacancy defects (e.g. Cu vacancies, Se vacancies) in the resistive switching film and the barrier properties at the electrode/resistive switching layer interface, it is expected to further enhance the device performance uniformity including On/Off ratio stability and cycle endurance, and improve its environmental adaptability and operational robustness. Second, existing research has mostly focused on single‑device performance optimization; there is an urgent need to develop low‑crosstalk array structure designs and compatible integration processes to address key issues such as low array yield and signal crosstalk. Third, novel neural network algorithms and architectures (e.g. spiking neural networks) that deeply match the ‘nonvolatile storage‑computation fusion’ characteristic of memristors should be developed to avoid the power loss of traditional algorithms on hardware, thereby accelerating the path toward practical applications.
